# The impact of integrated prevention and treatment on child malnutrition and health: the PROMIS project, a randomized control trial in Burkina Faso and Mali

**DOI:** 10.1186/s12889-017-4146-6

**Published:** 2017-03-09

**Authors:** Lieven Huybregts, Elodie Becquey, Amanda Zongrone, Agnes Le Port, Regina Khassanova, Lazare Coulibaly, Jef L. Leroy, Rahul Rawat, Marie T. Ruel

**Affiliations:** 1 International Food Policy Research Institute, 2033 K Street, NW, Washington, DC 20006 USA; 2 Helen Keller International Burkina Faso country office, Ouagadougou, Burkina Faso; 3 Helen Keller International Mali country office, Bamako, Mali

**Keywords:** Child malnutrition, Small quantity lipid-based nutrient supplement, Behavior change communication, Prevention, Research protocol

## Abstract

**Background:**

Evidence suggests that both preventive and curative nutrition interventions are needed to tackle child acute malnutrition (AM) in developing countries. In addition to reducing the incidence of AM, providing preventive interventions may also help increase attendance (and coverage) of AM screening, a major constraint in the community-based management of child acute malnutrition (CMAM) model. There is a paucity of evidence-based strategies to deliver integrated preventive and curative interventions effectively and affordably at scale. The aim of the Innovative Approaches for the Prevention of Childhood Malnutrition (PROMIS) study is to assess the feasibility, quality of implementation, effectiveness and cost-effectiveness of an integrated child malnutrition prevention and treatment intervention package implemented through a community-based platform in Mali and a facility-based platform in Burkina Faso.

**Methods/Design:**

The PROMIS intervention entails a comprehensive preventive package offered on a monthly basis to caregivers of children, while children are screened for acute malnutrition (AM). The package consists of behavior change communication on essential nutrition and hygiene actions, and monthly preventive doses of small quantity lipid-based nutrient supplements (SQ-LNS) for children aged 6 to 23.9 months. Positive AM cases are referred to treatment services offered by first-line health services according to the CMAM model.

The PROMIS intervention will be evaluated using a mixed methods approach. The impact study encompasses two types of study design: i) repeated cross-sectional surveys conducted at baseline and at endline after 24 months of program implementation and ii) a longitudinal study with a monthly follow-up for 18 months. Primary study impact measures include the incidence and endpoint prevalence of AM, AM screening coverage and treatment compliance. A process evaluation will assess the feasibility and quality of implementation of the intervention guided by country specific program impact pathways (PIPs). Cost-effectiveness analysis will assess the economic feasibility of the intervention.

**Discussion:**

The PROMIS study assesses the effectiveness of an innovative model to integrate prevention and treatment interventions for greater and more sustainable impacts on the incidence and prevalence of AM using a rigorous, theory-based randomized control trial approach. This type of programmatic research is urgently needed to help program implementers, policy makers, and investors prioritize, select and scale-up the best program models to prevent and treat AM and achieve the World Health Assembly goal of reducing childhood wasting to less than 5% globally by the year 2025.

**Trial registration:**

Clinicaltrials.gov NCT02323815 (registered on December 18, 2014) and NCT02245152 (registered on September 16, 2014)

**Electronic supplementary material:**

The online version of this article (doi:10.1186/s12889-017-4146-6) contains supplementary material, which is available to authorized users.

## Background

Globally, an estimated 52 million children suffer from acute malnutrition (AM) [1]. AM dramatically increases the risk of death: compared to well-nourished children, children with moderate acute malnutrition (MAM, i.e. weight-for-length z-score (WLZ) between -3 and -2 SD) are 3.4 times more likely to die and this goes up to a staggering 11.6 times in children with severe acute malnutrition (SAM, i.e. WLZ < -3 SD). Globally, AM or wasting is the underlying cause for an estimated 875,000 deaths of children under 5 years of age, representing about 12.6% of all deaths in this age group [1, 2].

The landscape for effective treatment of child SAM has changed dramatically in the last 10 years. This is due to the advent of Ready-to-Use Therapeutic Foods (RUTF) to treat SAM, a high-energy, lipid-based micronutrient-fortified supplement that can be easily used in field conditions, and the adoption of Community-based Management of Acute Malnutrition (CMAM). Under CMAM, children with SAM (but without complications) are treated at home with support from the primary health care facility [3]. Recently, the CMAM model was expanded to include the ambulatory treatment of MAM cases using Ready-to-Use Supplementary Foods (RUSF) or fortified flour blends [4]. Although the World Health Organization strongly recommends the CMAM model [3, 5], its effectiveness remains suboptimal when scaled up due to a combination of low coverage of screening and of low participation in seeking and completing treatment schedule [6]. Novel strategies that can increase CMAM coverage are therefore highly warranted.

As is the case for many health issues, preventing child malnutrition is more effective than treating it [7]. Approaches to prevent both acute and chronic child malnutrition include interventions to improve child nutrient intakes and foster optimal health, which in turn rely on households adopting optimal feeding, health, and care practices [8]. In many resource-constrained environments, however, households lack the means to ensure a nutritionally adequate diet for infants and young children. Recently developed specialized products, such as multiple micronutrient powders (MNPs) and small quantity lipid-based nutrient supplements (SQ-LNS), provide an opportunity to combine behavior change communication (BCC) that promotes adequate nutrition, health and care practices with access to products that help food-insecure households provide a nutritionally adequate diet to their young children [9, 10]. Different program models combining BCC and specialized products, and aimed at preventing child malnutrition (usually during the first 1000 days), have been developed and implemented. These preventive programs, however, have generally been implemented in parallel, rather than an integrated manner, with programs aimed at treating SAM. Evidence on how to integrate preventive services into treatment-focused programs such as CMAM and on the effectiveness of this approach is missing.

The aim of the Innovative Approaches for the Prevention of Childhood Malnutrition (PROMIS) multi-country study is to assess the feasibility, quality of implementation, effectiveness and cost-effectiveness of an integrated child malnutrition program that combines prevention and treatment interventions. The dual goal of the program is to increase coverage of screening for CMAM treatment, while also preventing new or repeated cases of AM. The intervention is implemented by Hellen Keller International (HKI) through a community-based platform in Senegal and Mali, and through a facility-based platform in Burkina Faso. PROMIS integrates a BCC intervention (promoting Essential Nutrition Actions (ENA) and Essential Health Actions (EHA)), the provision of SQ-LNS supplements for the prevention of child malnutrition, and the screening and referral of child AM cases. In Mali and Burkina Faso impact evaluations on the program’s effectiveness are conducted, whereas in Senegal only the feasibility of the program in peri-urban areas is assessed. The present paper focuses on the (cost-)effectiveness and process evaluation protocols for the studies in Mali and Burkina Faso only.

## Methods

### Study area

Mali and Burkina Faso, two landlocked countries situated in west Africa, are amongst the poorest countries in the world, ranking 179th and 183th on the Human Development Index, respectively [11]. Approximately 50 and 44% of Mali’s and Burkina Faso’s population live on less than US $1.90/day [12].

In Mali the study is conducted in the health districts of Bla and San situated in the Segou region in eastern Mali. The Bla health district has 28 Health Centers (HC), while San has 30 HC. Both health districts have one urban HC; the other HCs are situated in semi-urban or rural settings. In 2013, the prevalence of AM (defined in the Demographic and Health Surveys as WLZ < -2 SD) in children under five in the Segou region was comparable to the national average (12.9% vs 12.7%); at 3.5%, the prevalence of SAM (WLZ < -3 SD), however, was amongst the highest in Mali [13]. In Burkina Faso, the study is conducted in the Gourcy health district in the Northern region which includes 32 rural and one urban HC. In 2010 an estimated 16.5% of all children under five suffered from AM (WLZ < -2 SD) in this region, a prevalence comparable to the national average of 16.0%; the prevalence of SAM (WLZ < -3) was 7.2%, compared to the national average of 6.0% [14]. The study areas in both countries rely on rain-fed agriculture for food production; staple crops such as millet and sorghum are harvested once a year from September to December. Both study settings are malaria endemic areas with peak incidence during the annual rainy season from June to October [15].

### Study participants

In Mali, children between 6 and 23.9 months of age belonging to the catchment areas of 48 HCs are the target beneficiaries of the PROMIS intervention and, as such, constitute the study population. For logistical and budgetary reasons, we reduced the total number of HC clusters from 58 to 48, omitting the 10 most southern HC catchment areas in the San district. These 10 adjacent HC catchment areas were omitted because they were less accessible during the rainy season. In Burkina Faso the study population consists of children 0 to 17.9 months of age in the catchment areas of 32 rural HCs.

### The PROMIS intervention

The PROMIS intervention provides an integrated package that consists of two key components: 1) prevention (through age-stratified BCC targeted to the caregivers of the beneficiary children and the distribution of preventive doses of SQ-LNS), and 2) screening for, and referral of cases of AM. The feasibility and adequacy of several candidate platforms to deliver the program’s integrated package were assessed before the intervention started. The final selection made by HKI was based on their field experience and a quick feasibility assessment in each country. A comparison of the delivery platforms and the nutrition-related activities available in the intervention and control communities is provided in Table 1.

**Table 1:** Overview of PROMIS Program components supported by HKI in Mali and Burkina Faso

	Mali		Burkina Faso	
	Control	Intervention	Control	Intervention
PROMIS main delivery platform:				
- Monthly village gathering organized by CHV for children 6–23.9 months of age and their caregivers	X	X		
- Monthly WBC at HC for children 0–23.9 months of age and their caregiver			X	X
PROMIS main integrated package^a^ (received through PROMIS delivery platform):				
- Screening and referral for AM	X	X	X	X
- Nutrition, hygiene and health BCC in a large group			X	X
- Enhanced BCC on ENA/EHA in a small caregiver groups (organized by child age)	X	X		X
- SQ-LNS distribution and SQ-LNS related BCC		X		X
Other PROMIS activities (through various delivery platforms):				
- Capacity building supporting CMAM	X	X	X	X
- Support to quarterly screening and referral campaigns			X	X
- Radio programs on ENA/EHA topics	X	X	X	X
- Community theatre performance on ENA/EHA topics			X	X
- Village nutrition committee^b^			X	X

^a^The main integrated package corresponds to a group of services provided at the same time to the same beneficiaries, some as part of the national policy, others in addition to the national policy

^b^In each village, a village nutrition committee comprising 6 influential community members is set up to support BCC and for follow-up of relevant children

#### Mali: community-based platform

In Mali, HKI opted to implement the PROMIS intervention package through community health volunteers (CHV, *relais* in French). These community workers are present in every village in the study area and have as main responsibility the screening and referral of AM children within their neighborhood. HKI trained all CHV from intervention and control study groups in ENA/EHA related group BCC and set up a supply chain of SQ-LNS to every village in the intervention study area. CHV were asked to organize monthly village gatherings of caregivers with children between 6 and 23.9 months of age to deliver BCC and child AM screening sessions in all control and intervention villages throughout the project. In villages with more than 20 beneficiaries, two sessions (for caregivers of children 6–11.9 months and 12–23.9 months) are organized. Each monthly BCC session covers one ENA or EHA topic, which includes recommendations on breastfeeding, complementary feeding, nutrition during pregnancy and lactation, malaria prevention, nutrition during child illness, hygiene and the use of SQ-LNS. The BCC follows the Greet, Ask, Listen, Identify, Discuss, Recommend, Agree, set follow-up Appointment (GALIDRAA) communication approach [16]. At each session, children are screened for AM using mid-upper arm circumference (MUAC). Positive cases are referred to SAM or MAM treatment offered by first-line health services. BCC attendance, as well as the thematic topic discussed, and the result of the MUAC screening is recorded on the participant’s program card.

Conditional on their participation to the monthly village gathering, caregivers of beneficiary children from intervention villages receive a monthly supply of SQ-LNS (Nutriset, Malaunay, France) in 20 g sachets intended for daily use. The nutrient composition of these peanut-based multiple micronutrient-fortified supplements is provided in Table 2. The SQ-LNS serve two purposes: to improve micronutrient intake and to provide an incentive to attend the BCC and screening sessions. The distribution of SQ-LNS follows current guidelines on its use [17], including the promotion of continued breastfeeding.

**Table 2:** Composition of a daily dose (20 g) of SQ-LNS

Component	Amount per 20 g
Energy, kcal	118
Proteins, g	2.6
Lipids, g	9.6
Linoleic Acid, g	4.5
α-Linolenic Acid, g	0.5
Calcium, mg	280
Phosphorus, mg	190
Potassium, mg	200
Magnesium, mg	40
Zinc, mg	8
Copper, mg	0.34
Iron, mg	6
Iodine, μg	90
Selenium, μg	20
Manganese, mg	1.2
Vitamin A, mg	0.4
Vitamin B1, mg	0.3
Vitamin B2, mg	0.4
Niacin. mg	4.0
Pantothenic acid, mg	1.8
Vitamin B6, mg	0.3
Folic acid, μg	80
Vitamin B12, μg	0.5
Vitamin C, mg	30
Vitamin D, μg	5
Vitamin E, mg	6.0
Vitamin K, μg	30

#### Burkina Faso: health service-based platform

In Burkina Faso, the well-baby consultations (WBC, *consultation du nourrisson sain* in French) at the HC are used as the delivery platform. According to national policy, children between 1 and 12 months of age are expected to attend preventive WBCs at the HC on a monthly basis and then attend it every other month up to the age of 24 months. For PROMIS, this schedule was modified to include monthly visits for expanded to all children from 1 to 23.9 months old. Services delivered during these preventive visits include growth monitoring, vaccination, vitamin A supplementation and deworming. Caregivers also receive BCC as per national policy, which is delivered by CHW and/or HC staff in large groups often without a well-established curriculum. Caregivers in the intervention (but not in the control) clusters, receive additional BCC in smaller groups of 5 to 10 caregivers stratified by child age at the HC from either HC staff or CHW. This BCC follows a predetermined curriculum of ENA and EHA topics and is based on the GALIDRAA communication approach. The distribution of a monthly supply of SQ-LNS in the intervention group is limited to children 6 to 23.9 months of age; the distribution of the supplements serves the same dual role as in Mali—providing additional nutrients and incentive to participate in BCC and screening. HC staff record systematically anthropometry (weight, length and MUAC), and thus screen for AM, of all children that attend the WBC. Given the extra workload created by the PROMIS activities, community health workers (CHW) of the intervention group receive a small monetary incentive from HKI to support health staff in delivering the preventive package.

In both countries, HKI supports a number of other activities related to screening for and the prevention of AM. These activities take place outside the PROMIS delivery platforms, do not integrate screening and prevention, and are available to both treatment and intervention communities (Table 1). In both Mali and Burkina Faso, HKI supports the development of radio shows that sensitize the community on ENA/EHA topics. In Burkina Faso, HKI initiated theater plays on ENA/EHA topics; it supported the creation of village nutrition committees composed of 6 influential community members (2 respected elderly women, 2 CHW and 2 representatives of village authorities) to provide BCC on ENA/EHA, to screen children for AM and to promote adequate ENA/EHA practices; finally, it supports quarterly screening campaigns for AM in collaboration with other NGOs.

### Impact study

#### Study design

In both countries a two-arm cluster-randomized, non-masked, community-based, trial is used to estimate the effectiveness of the PROMIS intervention. The unit of randomization is the catchment area of the HC. PROMIS has the potential to reduce the prevalence of AM through two distinct pathways: 1) the preventive component (i.e. BCC and the consumption of SQ-LNS), which is expected to lower the incidence and consequently the prevalence of AM; and 2) the screening for AM component, which is expected to increase the number of cases identified, referred and treated. If early screening, referral and treatment are effective, it is expected that the it will shorten the duration of the episodes of AM and consequently also contribute to lowering its prevalence. To assess the importance of each pathway, the study combines a repeated cross-sectional and a longitudinal study design.

The repeated cross-sectional study design uses data collected at baseline and after 24 months of program implementation among a representative sample of children 0–17.9 months and 6–23.9 months of age in Burkina Faso and Mali, respectively. The cross-sectional study design allows us to assess the impact of the program on the prevalence of MAM and SAM in the full age range targeted by the program. In addition, the cross-sectional data will be used to assess the impact of the program on stunting, on the coverage of screening for AM, and on maternal knowledge and practices related to the BCC topics emphasized in the study (maternal and child health, nutrition and IYCF, and water, sanitation and hygiene).

The longitudinal study recruited individual children at 0–1.4 months (Burkina Faso) and 6–6.9 months of age (Mali). In each country, cohort children are followed monthly for 18 months to assess the impact of the preventive component of the program on the incidence of AM. Additionally, the longitudinal design is used to assess the impact of the program on child morbidity and to document changes in caregivers’ ENA/EHY knowledge and practices.

#### Study outcomes

Primary study outcomes for the cross-sectional study design in Mali and Burkina Faso are:

i) the prevalence of AM defined by a WLZ < -2 or a MUAC < 125 mm (only in children older than 6 months) or the presence of bilateral pitting edema;
ii) AM screening coverage defined as the number of children screened in the month preceding the survey (as reported by the caregiver) over the total number of eligible study children;
iii) AM treatment compliance defined as the number of AM children under appropriate treatment at the time of the survey over the total number of AM cases identified in the study sample.

Secondary study outcomes for the cross-sectional study include anthropometric outcomes mean WLZ, MUAC and Length-for-Age Z-score(LAZ), the prevalence of child stunting (LAZ < -2 SD) and severe stunting (LAZ < -3 SD), the prevalence of SAM (defined by WLZ < -3 SD, a MUAC < 115 mm (children older than 6 months), or bilateral pitting edema)); mean hemoglobin (Hb) concentration, child anemia (Hb < 11 g/dL) and severe anemia (Hb <7 g/dL); caregivers’ ENA and EHA and IYCF knowledge and practices.

For the longitudinal study, primary outcomes in both countries are

i) the incidence of AM (same definition of AM as above);
ii) monthly AM screening coverage (the number of children screened each month over the total number of eligible study children);
iii) AM treatment compliance (the number of AM children adhering to weekly or bi-weekly treatment until discharged over the total number of AM children that were scheduled for treatment).

Secondary outcomes include the relapse rate after treatment of MAM and SAM (number of MAM or SAM cases detected after being successfully discharged from MAM or SAM treatment), ponderal and linear growth (monthly WLZ and LAZ increment, respectively), incidence of stunting (LAZ < -2 SD), MUAC gain (MUAC increment per month), longitudinal prevalence (ie. number of days of illness divided by the total number of days of observation for each child) of child morbidity (acute respiratory infections, fever, diarrhea, vomiting, and malaria), patterns of child morbidity prevalence over time, caregiver’s knowledge and practices related to IYCF and ENA/EHA.

#### Sample size

We used Hayes and Bennet’s formulas to calculate the necessary sample sizes for the repeated cross-sectional and the longitudinal study [18]. For the repeated cross-sectional study design, we assumed a coefficient of inter-cluster (i.e. between HC) variation k of 0.25, a non-response rate of 15%, a type I error of 5% and a statistical power of 80%. In Mali we found that with an average cluster (i.e. HC catchment area) size of 48 children, 48 clusters (i.e. an overall sample size of 2304 children) will allow us to detect a decrease in the prevalence of AM of 5.3 percentage points assuming a baseline prevalence of 18.0%. This sample size allows us to detect a difference in screening coverage and treatment compliance of 6.7 percentage points between study groups assuming baseline values of 25% for both outcomes. In Burkina Faso, 32 rural clusters with an average cluster size of 72 children (2304 children total), will allow us to detect a decrease in the prevalence of AM of 5.4 percentage points assuming a baseline prevalence of 16.0%. This sample size allows us to detect a difference in screening coverage and treatment compliance of 7.5 percentage points between study groups assuming baseline values of 25% for both outcomes.

The longitudinal study is conducted in the same villages as the cross-sectional survey to maximize comparability. We assumed a coefficient of inter-cluster variation k of 0.2, a dropout rate of 20%, a type I error of 5% and a statistical power of 80%. In Mali, with 24 children in each of the 48 clusters (1152 children total) we can detect a 23.5% reduction in the incidence of AM, assuming a baseline incidence of 0.61 case per child-year. For Burkina Faso, 66 children in each of the 32 available clusters (total sample size of 2112 children) will allow us to detect a 23.5% change in the incidence of AM assuming a baseline incidence of 0.52 cases per child-year.

#### Randomization, sampling and study planning

In Mali, we applied stratified random allocation of the HC catchment areas to control and intervention study groups. HCs are governed by their own community health association and operate autonomously. They are thus expected to be quite heterogeneous in terms of organization and performance [19]. Stratifying clusters prior to randomization ensured a more balanced distribution of cluster-level covariates between study arms. We first stratified the HCs by hierarchical clustering using a set of criteria that are detailed in Table 3. Based on the visual inspection of the obtained cluster dendrogram obtained from hierarchical clustering with complete linkage, we subdivided the clusters into 3 strata for the Bla district and 2 strata for the San district. Random allocation to control or intervention groups was conducted within each stratum during a community ceremony in Bla and San in the presence of local health authorities. Forty-eight identical pieces of paper with either ‘control’ (*n* = 24) or ‘intervention’ (*n* = 24) written on them were mixed in a bag by the project coordinator of HKI. Each HC director drew one piece of paper thus allocating his HC catchment area to the control or intervention study group.

**Table 3:** Criteria used to stratify HC catchment areas in Mali

Criteria
- Type of staff working in HC
- Accessibility during rainy season
- Type of the catchment area (urban/semi-urban/rural)
- Number of villages covered
- Number of villages with community health workers (CHW)
- Vaccination coverage
- Total number of children 6-23.9 months
- Proportion MAM admissions/total population
- Proportion SAM admissions/total population
- Distance between villages and HC
- Distance between HC and District hospital

In Burkina Faso, simple (i.e. non-stratified) random allocation was used. As in Mali, randomization took place at a community event in Gourcy with local health authorities. Thirty-two identical pieces of paper with either ‘control’ (*n* = 16) or ‘intervention’ (*n* = 16) written on them were mixed in a bag for randomization of the 32 rural HC. The allocation of the urban HC was conducted separately by drawing a piece of paper from a bag containing 2 pieces of paper (1 ‘control’ and 1 ‘intervention’). The primary impact analysis in Burkina Faso will be limited to the 32 rural HC catchment areas since the services offered and the population served in the urban HC are not comparable to those at the rural HCs.

In each country, a census to identify infants and pregnant women was organized prior to the start of the cross-sectional surveys and prior to the beginning of the longitudinal study. In Mali, we excluded villages and small settlements with a population of less than 300 to lower the logistical cost. In Burkina Faso, when the HC catchment area included more than 3 villages, we randomly selected 3 villages (using probability proportional to population size sampling) and then randomly selected an equal number of households with an eligible child using Stata. Since age is an important predictor of AM and nutrition and health related practices, we stratified children in three equal age groups (Mali: 6–11 m; 12–17 m; 18–23 m and Burkina Faso: 0–5 m; 6–11 m; 12–17 m) and drew a random sample from each age group for the cross-sectional study.

Inclusion criteria for the cross-sectional study were: i) a child 6–23.9 months (Mali) or 0–17.9 months of age (Burkina Faso); ii) child’s main caregiver living the study area since the child’s delivery; iii) child without congenital malformations that hinder the measurement of anthropometry. The inclusion criteria for the longitudinal study were: i) a child 6–6.9 months (Mali) or 0–1.4 months of age (Burkina Faso); ii) child not being in a state of AM; iii) child’s main caregiver living the study area since the child’s delivery; iv) child without congenital malformations that hinder the measurement of anthropometry.

The baseline cross-sectional surveys were conducted between November-December 2014 and February-March 2015 in Burkina Faso and Mali, respectively. Endline surveys are organized 24 months later during the same calendar months in both countries. The longitudinal study recruited eligible children between March and August 2015 in Burkina Faso and between July and September 2015 in Mali. Cohort children are followed up monthly for 18 months. The last follow-up measurements for the longitudinal study are foreseen for February 2017 in Burkina Faso and April 2017 in Mali.

#### Questionnaires

**Fig. 1:**
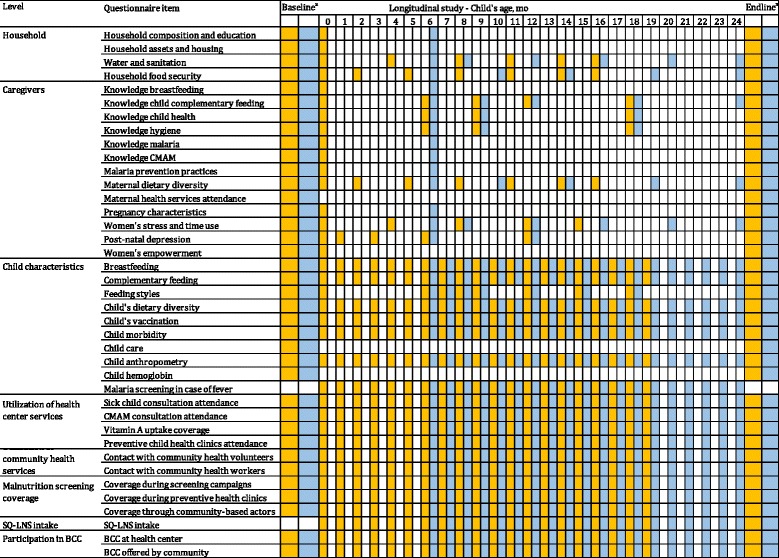
Household questionnaire items and chronogram of measurements for PROMIS Mali (*light blue*) and Burkina Faso (*orange*); ^a^baseline and endline from the repeated cross-sectional surveys

Data are collected at the HC, community, household, and individual level using questionnaires. HC staff questionnaire (administered as part of the cross-sectional survey) assesses, among others, the organization of preventive and CMAM consultations, staff occupancy, HC facilities, stock turnover of medicines and therapeutic foods. The community health worker questionnaire (also administered as part of the cross-sectional survey) focuses on the screening, referral and follow-up activities for MAM and SAM cases in the community. Both the health staff and CHW questionnaires further assess ENA, EHA and CMAM knowledge as well as job satisfaction, supervision and training received. Household questionnaires were developed for the cross-sectional and longitudinal studies. Figure 1 gives an overview of the questionnaire themes and the timing of administration. All survey data are collected through a Computer-assisted personal interviewing format (CAPI) designed using Surveybe version 4 and 5 software (Surveybe, UK).

#### Measurement instruments

The child’s weight is measured to the nearest 100 g using an electronic scale (SECA 876, Germany). Length is measured to the nearest 1 mm using a length board (Weigh and Measure LCC, USA). Mid-upper arm circumference measurements are obtained using a non-stretchable tape with 0.1 cm precision (SECA 201, Germany). All measurements are taken in duplicate by an anthropometrist and an assistant. Maternal weight and height are recorded using scales (SECA 877) and stadiometers (Weigh and Measure LCC, USA) respectively. WLZ and LAZ scores will be calculated using the 2006 World Health Organization growth reference [20]. All anthropometrists were standardized before the fieldwork started against measurements of a lead anthropometrist and are re-standardized every 2 months throughout the longitudinal study [21]. Children that are diagnosed with AM are referred to the nearest health center where treatment is offered free of charge.

A 3-day recall period is used for infant morbidity (acute respiratory infections, diarrhea, fever and malaria). This recall period leads to more accurate estimates at the expense of a small to moderate loss of statistical power as compared to a 7-day recall period, especially if morbidity signs are common, the number of measurements per individual exceeds 10 or 12, and in cluster-randomized trials [22]. A diarrheal episode is defined by at least 3 liquid or semi-liquid stools in the last 24 h. Fever is assessed in two ways. First, it is measured using a standard thermometer. If no fever is recorded, fever episodes are assessed through 24 h recall. The presence of acute respiratory infection (ARI) is defined as presence of specific ARI-related symptoms being cough, difficulty breathing, grunting, rapid breathing, using a 3-day recall. Malaria is diagnosed using a fingerpick rapid diagnostic test in cases where the child’s axillary temperature is over 37.5 °C or the mother reports fever in the last 24 h. Child Hb concentration in capillary blood is measured by spectrophotometry using a portable HemoCue device (HemoCue Ltd, Dronfield, United Kingdom). Children that are diagnosed with severe anemia or malaria are referred to the nearest HC for treatment. For all such cases the project covers the charges related to consultation and treatment.

#### Data management and analysis

The repeated cross-sectional study design will be used to estimate program impact after 2 years of program implementation. Linear and linear probability mixed-effect regression models will be used for continuous and binary outcomes respectively. Although the randomization is expected to minimize average differences at baseline between groups, we will adjust regression models for baseline values of the outcome of interest and covariates that should not have changed as a consequence of the intervention to improve the precision of the estimates. Regression models will further be adjusted for clustering at HC catchment area level. Explorative analysis will assess effect modification by HC and CHW characteristics by testing interaction terms and if statistically significant at 10% level considered for sub-group analysis.

The monthly measurements of child weight, MUAC and WLZ increments, and linear growth (length and LAZ increments) in the longitudinal study will be modeled using linear mixed-effects models which will include an interaction term between follow-up time and intervention allocation to assess the intervention’s impact on these outcomes over time. All longitudinal analysis models will further be adjusted for child sex, child age, primiparity, the baseline value of the outcome of interest, covariates that should not have changed as a consequence of the intervention, stratum of HC catchment areas (in Mali). Interactions between intervention and baseline covariates will be inspected and if statistically significant at 10% level considered for sub-group analysis.

For the analysis of AM incidence and the longitudinal prevalence of morbidity, we will use mixed-effects Poisson regression models with robust estimation of standard errors. The mixed-effects models will be adjusted for clustering by HC catchment area and individual to estimate the correct standard errors. Data will be analyzed on intention-to-treat basis.

We plan a causal mediation analysis using the longitudinal data to identify the pathways of impact on the primary study outcomes. Multiple imputation of missing data will be conducted to assess the robustness of the findings.

Data management, data cleaning and statistical analyses will be done using Stata 14.1 (Statacorp, USA). The statistical significance will be set at 5%. All statistical tests will be two-sided.

### Cost and cost-effectiveness

Costs are estimated using a combination of ingredients based and Activity-Based Costing methods (ABC-I). The ABC-I method defines all the activities associated with program implementation and assesses the cost of all of the ingredients (i.e. inputs) used to achieve each activity. Costs are measured as the economic costs (i.e. taking a societal perspective) and not just as the accounting cost. The costs of volunteer labor, in-kind donations, and household time, for instance, are included in the total cost. The study thus includes all relevant costs incurred by institutions and communities [23–25], but will also assess the opportunity costs made by mothers to participate in the program. Data on costs and the allocation of resources across activities were collected through reviewing the programs’ accounting records and documentation, and through key informant interviews and focus group discussions with relevant program implementation staff.

Analysis will focus on the total program costs, the incremental costs of the additional activities in the intervention area, and will disaggregate by costs for preventive versus treatment interventions. Cost-effectiveness ratios will be calculated using costs and the impact on the primary outcomes of the intervention. Incremental cost-effectiveness ratios will be calculated by dividing the additional costs in the intervention area by the additional number of cases of AM averted and the change in the prevalence of AM at endpoint in the intervention area (as compared to the control area). This ratio represents the additional cost to prevent one additional case of AM or to reduce AM prevalence by one percentage point. Comparison with other similar published work will be considered where appropriate.

### Process evaluation

The overall objective of the process evaluation is to assess the feasibility, quality of implementation and service delivery, and to identify operational and utilization constraints. The process evaluation examines the primary inputs, processes, outputs, and outcomes along the program impact pathway in order to determine the “how” and “why” of program impact. The specific objectives of the process evaluation, along with the corresponding study methods and the study population or program delivery points studied in each country are detailed in Table 4.

**Table 4:** Process evaluation objectives, research methods, and program delivery point or study population by program level

Program Level	Objectives	Research Method	Program Delivery Point or Study Population
Program Implementation	• Describe the actual implementation of the program and identify potential bottlenecks	Semi-structured continuous observations of program activities	*Burkina Faso*: WBC *Mali*: Monthly village gathering/point of distribution of SQ-LNS
	• Understand key stakeholders’ (frontline health agents) perceptions of specific aspects of the program • Understand how the program is integrated into the existing health system • Describe how the program contributes to the implementation of the national protocol • Describe how the program adds to the frontline health agents’ workload	Individual semi-structured qualitative interviews	*Burkina Faso*: Health staff; community health workers involved in the implementation of the program at the level of the HC *Mali*: CHVs directly involved in the implementation of the program
Household & Individual Beneficiary Utilization	• Understand mothers’ perception of the program, perceived incentive structure and individual costs of receiving the program • Understand mothers’ barriers and facilitators to participate in the program • Understand the perception of mothers of the quality of services at the CSPS/CSCOM • Understand how mothers translate their knowledge into practice	Individual semi-structured qualitative interviews	*Burkina Faso & Mali*: Mothers of children who are eligible for the program
	• Describe individual use of SQ-LNS • Identify salient program messages for mothers	Group free listing	*Burkina Faso*: Mothers of children who have attended WBC *Mali*: Mothers of children who have attended monthly group BCC/SQ-LNS distribution

Since the design of a rigorous process evaluation requires a solid understanding of the program impact pathway (PIP), the first step was to develop a PIP in both countries [26]. We reviewed program documents and consulted with program designers, implementers, and managers to be sure the intended program implementation and outcomes were appropriately represented. The PIP documents the institutions implicated in the program; the actors involved in the program; the flow of program inputs through those institutions and actors; the sequencing of events; and the envisioned effects of the program.

A combination of random and purposive sampling techniques was used for the process evaluation. Three focused ethnographic data collection methods were used: semi-structured continuous observations of program activities, semi-structured individual qualitative interviews with implementation staff and beneficiary mothers, and group free listing with beneficiary mothers. *Semi*-*structured continuous observation* is a technique where the researcher observes activities of a participant or a set of participants directly without engaging in those activities or interrupting to ask questions. *Individual semi*-*structured qualitative interviews* consist of questions and probes that are open-ended, but are guided by a set of topics and questions to be discussed between the interviewer and one respondent. These semi-structured interviews are intended to illuminate the “why” and “how” and provide depth of understanding of a particular issue. *Free listing* is used to generate data that describes how items within a discrete domain (with list-able content) are categorized within a particular group of people; the order in which the item falls in the list, and the number of times an item appears across multiple lists represents the “saliency” of the item [27–29]. In analyzing the frequency of messages that beneficiary mothers report receiving as part of the program, we will be able to determine which program messages are most salient for these mothers. The breadth of a particular domain can also be illustrated with free listing, generating a wide range of items that constitute a domain for that specific group [27–29]. In this study, we use free listing to describe the variety of ways in which SQ-LNS was fed to the child (or by other people).

Data are recorded by hand (semi-structured continuous observation, free listing) and by digital recorders (semi-structured interview, free listing). A narrative summary is written by the enumerator conducting the semi-structured continuous observation immediately following data collection. Digitally recorded semi-structured interview data are transcribed and translated simultaneously from the local language into French by bilingual transcribers. For the free listing, the digital recording served to check the data collected by hand.

Semi-structured continuous observation data are coded using grounded theory (i.e., the researcher codes data without a pre-defined code list and mark themes as they emerge in the data). Semi-structured interview data are coded using list of codes developed a priori based on the research questions and themes of interest. All coding utilizes NVivo v.11 software. Coded output will be summarized by themes that correspond to the specific process evaluation research objectives. Free listing data are coded by category of interest and analyzed using basic descriptive statistics.

## Discussion

Child AM continues to be a major global health problem [2]. The answer to this challenge lays in the effective implementation of the CMAM model ensuring sufficient screening and treatment coverage of AM cases. Integrated programs that work both on the prevention and curative side of undernutrition hold the potential to reduce the prevalence of AM by reducing its incidence and enhancing its treatment effectiveness. Evidence on how to successfully integrate preventive services into treatment programs for AM however is missing. As a consequence, nothing is known about the effectiveness of this approach.

The Innovative Approaches for the Prevention of Childhood Malnutrition (PROMIS) seeks to fill these knowledge gaps and uses a rigorous, theory-based randomized control trial approach to assess the effectiveness of an innovative model to integrate prevention and treatment interventions for greater and more sustainable impacts on the incidence and prevalence of AM. The study’s cross-sectional and longitudinal components will allow us to disentangle the program’s effect on the incidence and prevalence of child AM. The evaluation also includes a strong process evaluation component that will help document implementation successes and challenges, including on delivery and quality of the programs as well as on utilization by targeted beneficiaries. The costing and cost-effectiveness components will provide rich data on another neglected area—information on how much it would cost to accelerate progress in reducing AM. This type of evidence is urgently needed to help program implementers, policy makers, and investors prioritize, select and scale-up the best program models to prevent AM and achieve the World Health Assembly goal of reducing childhood wasting to less than 5% globally by the year 2025.

## Additional file

Additional file 1: SPIRIT Checklist. (DOCX 37 kb)
Additional file 2: WHO Trial Registration elements. (DOCX 37.2 kb)

## Abbreviations

ABC-I: Activity-based costing-ingredients
AM: Acute malnutrition
BCC: Behavior change communication
CHW: Community health workers
CMAM: Community-based Management of Acute Malnutrition
EHA: Essential hygiene actions
ENA: Essential nutrition actions
GALIDRAA: Greet, ask, listen, identify, discuss, recommend, agree, set follow-up appointment
Hb: Hemoglobin
HC: Health Center
HKI: Helen Keller International
IYCF: Infant and Young Child Feeding
LAZ: Length-for-age Z-score
MAM: Moderate acute malnutrition
MUAC: Mid-upper arm circumference
PIP: Program impact pathway
SAM: Severe acute malnutrition
SQ-LNS: Small quantity lipid based nutrient supplement
WLZ: Weight-for-length Z-Score
