# Supplementary material for: The impact of integrated prevention and treatment on child malnutrition and health: the PROMIS project, a randomized control trial in Burkina Faso and Mali
Source: BMC Public Health. 2017 Mar 9;17:237. doi: 10.1186/s12889-017-4146-6 (PMC5343313; doi:10.1186/s12889-017-4146-6)
Supplement: Additional file 2: — WHO Trial Registration elements. (DOCX 37.2 kb) [file 12889_2017_4146_MOESM2_ESM.docx]

**Extended information. Study Protocol: The effect of integrated prevention and treatment on child malnutrition and health: the PROMIS project, a randomized control trial in Burkina Faso and Mali**

Trial registration

**WHO Trial Registration Data Set**

| **Data category** | **Information** |
| --- | --- |
| Primary registry and trial identifying number | ClinicalTrails.gov NCT02323815 (Mali) and NCT02245152 (Burkina) |
| Date of registration in primary registry | December 18, 2014 (NCT02323815)  September 16, 2014 (NCT02245152) |
| Secondary identifying numbers | - No 2014/110/CE/FMPOS (Ethics committee of University of Bamako) for PROMIS Mali - No 2014-9-113 (National Ethics committee of Burkina Faso) for PROMIS Burkina Faso |
| Source(s) of monetary or material support | Global Affairs Canada |
| Primary sponsor | International Food Policy Research Institute (IFPRI) |
| Contact for public queries | Lieven Huybregts ([l.huybregts@cgiar.org](mailto:l.huybregts@cgiar.org)) and Elodie Becquey ([e.becquey@cgiar.org](mailto:e.becqueye@cgiar.org)) |
| Contact for scientific queries | Lieven Huybregts ([l.huybregts@cgiar.org](mailto:l.huybregts@cgiar.org)) and Elodie Becquey ([e.becquey@cgiar.org](mailto:e.becquey@cgiar.org)) |
| Public title | Study Protocol: The effect of integrated prevention and treatment on child malnutrition and health: the PROMIS project, a randomized control trial in Burkina Faso and Mali |
| Scientific title | Study Protocol: The effect of integrated prevention and treatment on child malnutrition and health: the PROMIS project, a randomized control trial in Burkina Faso and Mali |
| Countries of recruitment | Mali and Burkina Faso |
| Health condition(s) or problem(s) studied | Child acute malnutrition (AM) |
| Intervention(s) | **Mali**: PROMIS intervention package consisting of small quantity-lipid based nutrient supplements (SQ-LNS), AM screening and tailored behavior change communication (BCC) on essential nutrition actions (ENA) and essential hygiene actions (EHA)versus AM screening tailored BCC  **Burkina Faso**: PROMIS intervention package consisting of SQ-LNS, AM screening and tailored BCC on ENA/EHA versus standard BCC (per national policy) and AM screening. |
| Key inclusion and exclusion criteria | Ages eligible for study: 6-23.9m in Mali and 0-17.9m in Burkina Faso |
|  | **Cross-sectional study (baseline and endline)**  Inclusion Criteria:   - - At least one index child 6-23.9 and 0-17.9 months of age in the household in Mali and Burkina Faso respectively; - - Mother should be living in the study area since the index child's delivery; - - Singleton infants.   Exclusion Criteria:   - - Index child should not present congenital deformations that hamper anthropometric measurements   **Longitudinal study**  Inclusion Criteria:   - - Child 6-6.9 and 0-1.4 months of age in Mali and Burkina Faso respectively; - - Child with WHZ>-2 and MUAC>125 mm (only in children above 6 months of age) and no bilateral pitting edema; - - Mother should be living in the study area since the index child's delivery; - - Singleton infants   Exclusion Criteria:   - - Congenital malformations that make anthropometric measurements impossible - - Mother planning to leave the study are in the coming year |
| Study type | Interventional |
|  | Allocation: Cluster randomized controlled trial with two evaluation designs:   1. Repeated cross-sectional surveys (baseline-endline) 2. Longitudinal study |
|  | Primary purpose: Prevention and Treatment |
| Date of first enrolment | Mali: January 30, 2014  Burkina Faso: October 6, 2014 |
| Target sample size | Mali (48 clusters)  Repeated cross-sectional surveys: n=2,304  Longitudinal study: n=1,152  Burkina Faso (32 clusters)  Repeated cross-sectional surveys: n=2,304  Longitudinal study: n=2,112 |
| Recruitment status | Recruiting |
| Primary outcome(s) | Primary study outcomes for the cross-sectional study in Mali and Burkina Faso are i) the prevalence of AM defined by a WLZ<-2 or a MUAC< 125mm (only in children older than 6 months) or the presence of bilateral pitting edema; ii) AM screening coverage defined as the number of children screened in the month preceding the survey (as reported by the caregiver) over the total number of eligible study children; iii) AM treatment compliance defined as the number of AM children under appropriate treatment at the time of the survey over the total number of AM cases identified in the sample. AM was defined as WLZ<-2 (all ages), a MUAC< 125mm (only in children older than 6 months), or the presence of bilateral pitting edema (all ages). |
| Key secondary outcomes | Secondary study outcomes for the **cross-sectional study** are anthropometric outcomes like mean WLZ, MUAC and Length-for-Age Z-score(LAZ), the prevalence of child stunting (LAZ <-2 SD) and severe stunting (LAZ<-3 SD), the prevalence of SAM (WLZ<-3 SD, a MUAC< 115mm (children older than 6 months), or bilateral pitting edema), mean hemoglobin (Hb) concentration, child anemia (Hb < 11g/dL) and severe anemia (Hb <7g/dL), caregivers’ ENA and EHA knowledge, caregiver’s knowledge and practices related to Infant Young Child Feeding (IYCF) and ENA/EHA.  Secondary outcomes for the **longitudinal study** include the relapse rate after treatment of MAM and SAM (number of MAM or SAM cases detected after being successfully discharged from MAM or SAM treatment), ponderal and linear growth (monthly WLZ and LAZ increment, respectively), incidence of stunting (LAZ<-2 SD), MUAC gain (MUAC increment per month), longitudinal prevalence of child morbidity (acute respiratory infections, fever, diarrhea, vomiting, and malaria), morbidity pattern over time, caregiver’s knowledge and practices related to IYCF and ENA/EHA. |
